# Therapeutic dilemma in the management of a patient with the clinical picture of TTP and severe B_12_ deficiency

**DOI:** 10.1186/s12878-015-0036-2

**Published:** 2015-12-01

**Authors:** Kara Walter, Jennifer Vaughn, Daniel Martin

**Affiliations:** Department of Medicine, University of Washington, Seattle, WA 98195-7710 USA; Division of Hematology, Department of Medicine, University of Washington, Seattle, WA 98195-7710 USA; Seattle Cancer Care Alliance, 825 Eastlake Ave. E, Seattle, WA 98109 USA

**Keywords:** TTP, pseudo-TTP, Anemia, Pernicious anemia, Schistocytes, Cobalamin

## Abstract

**Background:**

Idiopathic thrombotic thrombocytopenic purpura (TTP) is a rare hematological emergency characterized by the pentad of microangiopathic hemolytic anemia, thrombocytopenia, neurological symptoms, renal injury, and fever that is invariably fatal if left untreated. Prompt intervention with plasma exchange minimizes mortality and is the cornerstone of therapy. Rare reports have described “pseudo-TTP” driven by extreme hematologic abnormalities resulting from deficiency of vitamin B_12_. Distinguishing between these entities can pose a diagnostic and therapeutic challenge.

**Case presentation:**

A 77 year old female presented with altered mental status, renal insufficiency, thrombocytopenia and evidence of microangiopathic hemolytic anemia, suggesting TTP. Workup demonstrated macrocytosis and reticulocytopenia, and B_12_ level was unmeasurably low. Other elements of her clinical presentation, including volume loss and bleeding suggested a multifactorial pathogenesis could be contributing to her laboratory abnormalities, reducing the likelihood that she had TTP. The risks and benefits of treating aggressively with therapeutic plasma exchange (TPE) for TTP were considered given the diagnostic possibilities. The patient received TPE initially, with rapid de-escalation after her clinical response suggested “pseudo-TTP” from B_12_ deficiency was the driving the process. B_12_ supplementation corrected her hematologic abnormalities and she remains well two years after presenting.

**Conclusions:**

TTP is a rare condition with fatal consequences if left untreated. Guidelines recommend TPE even if there is uncertainty about the diagnosis of TTP. B_12_ deficiency is common, though not typically associated with severe hematologic abnormalities. We compare the presenting characteristics of all thirteen cases of pseudo-TTP reported in the literature with those from patients in case series of TTP to suggest a set of parameters that can help clinicians distinguish between pseudo-TTP and TTP and guide decision making regarding intervention. Evaluation of all TTP cases should include a B_12_, methylmalonic acid level and reticulocyte count. Reticulocytopenia suggests B_12_ deficiency. Finally an LDH level above 2500 IU/L is relatively uncommon in TTP and should suggest consideration of B_12_ deficiency.

## Background

Idiopathic TTP (reviewed in [1]) is a rare hematological emergency (four cases per million) driven by an inhibitory autoantibody to the ADAMTS13 metalloproteinase causing the accumulation of unusually large von Willebrand factor multimers. These multimers cause platelet aggregation and thrombi resulting in end organ damage. TTP is characterized by the pentad of microangiopathic hemolytic anemia, thrombocytopenia, neurological symptoms, renal injury, and fever that is invariably fatal if left untreated. Prompt intervention with TPE minimizes mortality, with complete response rates of roughly 75 % [2, 3]. TPE, however, is also associated with considerable risks. A cohort study of 302 consecutive patients from the Oklahoma TTP-HUS registry over 15 years showed a 2.3 % mortality rate and a 24 % rate of major complications secondary to plasma exchange [4]. B_12_ deficiency (reviewed in [5]) is seen in 4 % of older adults with a median age range of 70 to 80, and is often missed due to its subtle clinical manifestations. The classic hematologic changes seen in B_12_ deficiency include macrocytic anemia and neutrophil hypersegmentation. Patients can present with leukopenia, thrombocytopenia, and elevated serum lactate dehydrogenase and bilirubin [6]. One study has shown that approximately 10 % of patients with symptomatic cobalamin deficiency have significant hematologic manifestations including pancytopenia, severe anemia, and microangiopathy [7]. It should also be noted that patients undergoing bariatric surgery are at risk for B_12_ deficiency and that the popularity of this procedure is growing. In 2008, roughly 220,000 bariatric surgeries were performed in the United States, a large fraction of which were gastric bypass procedures [8]. The prevalence of low vitamin B_12_ was found to be 11 % one year after year after roux-en-Y gastric bypass [9]. In this report we present a patient with a presentation concerning for TTP with clinical features illustrating potential clinical similarities between TTP and severe B12 deficiency.

## Case presentation

A 77 year-old female was brought to a neighboring hospital with altered mental status, having been found unresponsive at home with evidence of bowel incontinence and bloody diarrhea. The patient had been well until two weeks prior to admission when she developed symptoms of nausea, vomiting and diarrhea that progressively worsened until the day of admission.

On presentation the patient was confused, afebrile and moderately hypotensive. Initial hemoglobin was 55 g/L with a mean corpuscular volume of 120 fL. Platelet count was 40 × 10^9^/L and white blood cell count was 5.9 × 10^9^/L. Reticulocyte count was 3.4 % and corrected reticulocyte count 1.2 %. The peripheral blood smear, interpreted by the pathologist, showed anisocytosis, poikilocytosis, and multiple schistocytes. Hypersegmentation of neutrophils was not noted. Other pertinent laboratory findings included a creatinine of 300 μmol/L, lactate dehydrogenase (LDH) of 3981 IU/L, and INR >10 in the setting of warfarin use for a thrombosis four months prior to admission. Her fibrinogen level was 4.44 g/L. The patient received 6 units of platelets, 3 units of packed red blood cells, and 3 units of fresh frozen plasma and crystalloid support. She was treated empirically with intravenous pantoprazole. Her blood pressure normalized and mental status improved substantially although incompletely with these measures. A vena cava filter was placed. B_12_ levels drawn on admission returned undetectable and she was given 1 mg B_12_ intramuscularly. Twenty four hours after admission, she was referred to our hospital for emergent TPE for a presumptive diagnosis of TTP given her altered mental status, renal insufficiency, thrombocytopenia and evidence of microangiopathic hemolytic anemia.

**Fig. 1:**
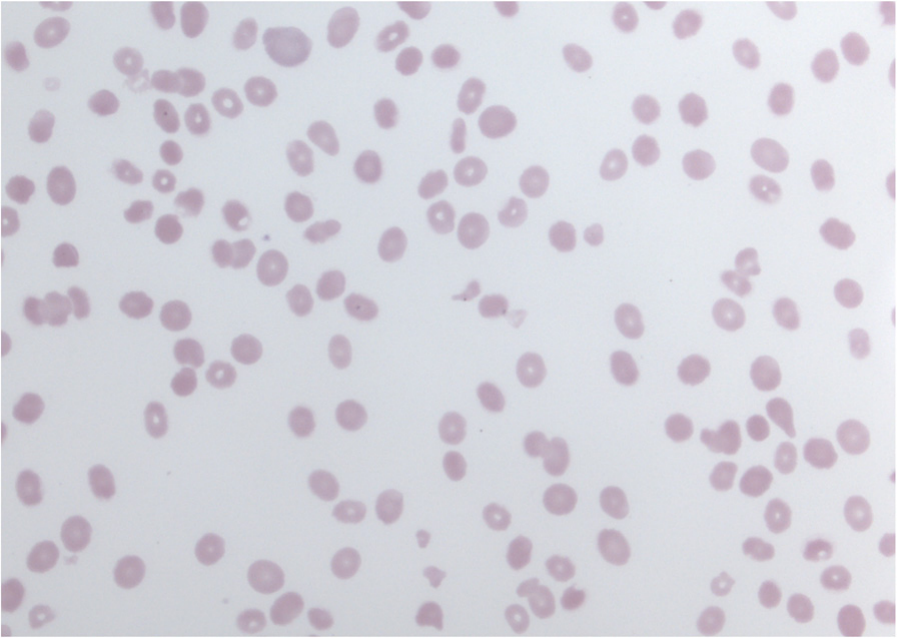
Peripheral smear (60×) showing anisopoikilocytosis with occasional teardrops cells and scattered schistocytes

Upon arrival her hemoglobin had improved to 99 g/L and platelet count to 60 × 10^9^/L. Repeat peripheral smear was notable for marked anisopoikilocytosis with occasional teardrops cells and scattered schistocytes Fig. 1. Haptoglobin was undetectable and LDH remained elevated at 3360 IU/L. A direct Coombs test was negative. Her INR was 1.3. Her creatinine had fallen to 152 μmol/L. The fractional excretion of sodium and urinary sediment were suggestive of pre-renal acute kidney injury/acute tubular necrosis.

The responsiveness of the patient’s platelet count to transfusion, improvement in renal function and plausibility of B_12_ deficiency as an explanation called into question the diagnosis of TTP. However, given the potential adverse consequences delayed plasma exchange in TTP, the decision was made to proceed with TPE. A double lumen apheresis catheter was placed and she received TPE for three consecutive days along with daily intramuscular cobalamin. During this time, her LDH fell dramatically, her mental status returned to baseline, and her creatinine continued to decline. However her hemoglobin and platelet count did not improve.

Given the lack of appreciable change in platelets and hemoglobin after three days of TPE, the decision was made to hold plasma exchange and observe. On hospital day five (six days after starting therapy with B_12)_, her platelet count began to rise and normalized on hospital day nine. LDH fell dramatically two days after starting therapy and stabilized at a slightly elevated level (~375 IU/L) after five days of therapy. Her creatinine continued to fall and reached a stable baseline of 57 μmol/L on hospital day ten. Anti-intrinsic factor antibody was later found to be positive. Her apheresis catheter was removed on hospital day seven. Upper endoscopy demonstrated atrophic gastritis. Colonoscopy showed left sided diverticulosis and internal hemorrhoids, thought to be the source of her bleeding at presentation. She was discharged after 12 days on enoxaparin and warfarin with the recommendation to remove the IVC filter in six months. Monthly B_12_ was recommended for life. Three weeks after discharge her hemoglobin was normal. She remains in good health greater than one year following discharge.

## Discussion

This patient presented with findings highly suspicious for TTP, but ultimately attributable to multiple coincident causes that combined to produce this clinical scenario. Her microangiopathic picture and thrombocytopenia were attributable to severe B_12_ deficiency and volume and blood loss from diarrhea and gastrointestinal bleeding (in the setting of supratherapeutic anticoagulation) caused acute kidney injury, and contributed to mental status changes. This case presented a dilemma in which the potential lifesaving benefit of prompt therapeutic intervention for TTP was balanced against the potential morbidity and mortality of the intervention itself, given the possibility of a masquerading processes. In this case the decision was made to incur the upfront risk of TPE to avoid the possibility of harm through delay of therapy for TTP. Ongoing TPE was made contingent on evidence of response or non-response.

In review of the literature, our patient is the fourteenth described with B_12_ deficiency mimicking TTP and the first where acute kidney failure complicated the differential diagnosis. Prior descriptions have used the term pseudo-TTP to describe this clinical scenario. The characteristics of six patients individually described in case reports are summarized in Table 1. Seven other patients have been described in a case series from a French referral center seen over a 10 year period [10, 11] with data aggregated. The B_12_ deficient patients seen in this series had an average platelet count of 73 × 10^9^/L, ranging from 38 × 10^9^/L to 130 × 10^9^/L. They were all reticulocytopenic, with absolute reticulocyte counts between 6.3 to 39.8 (10^9^/L). The average LDH level was 7310 IU/L (range 1084–16,520 IU/L). Mean data is presented in Table 1. The B_12_ levels ranged from 15 to 111 umol/l in this series.

**Table 1:** Characteristics of pseudo-TTP patients individually reported in the literature and averages from a case series

Case	Year	Sex	Age	WBC (×10^9^/L)	HCT	HGB	PLT (nadir) (×10^9^/L)	LDH (IU/L)	MCV (fL)	Retic × 10^9^/L	TPE	Ref
1	1998	F	38	3.6		39	260	5700	102	20	NO	[22]
2	1999	F	68	3.2		32	110	7900	112	34	YES	[23]
3	2003	M	38	2.2		45	50	19,384	90	10	YES	[24]
4	2008	M	48	6.3		50	380	8988	80	13	YES	[14]
5	2009	M	52	3.6	27		960	4604	107	31	NO	[25]
6	2011	F	31	4.2		57	420	4579	110	NR	YES	[26]
Mean values from seven patients												
Series			72	3.4		42	73	7310	111	13		[10]

Differentiating pseudo-TTP from TTP is potentially challenging even if both are under consideration. A number of parameters including renal function, MCV, neutrophil hypersegmentation, patient age, and LDH can alter the pretest probability for either diagnosis. Review of the reported cases in comparison to data from TTP case series can provide some important “lessons learned” and guidelines for workup of patients with TTP. None of the 13 cases reported prior to ours described renal failure. However, renal failure is seen in only approximately one half of patients in case series of TTP [12, 13]. An elevated MCV should raise the possibility of B_12_ deficiency. However, the MCV was in the normal range in three of the reported cases, presumably due to the effect of schistocytes in calculating the MCV. Neutrophil hypersegmentation is frequently seen in B_12_ deficiency, and should raise the suspicion for a nutritional deficiency [6]. The measurement of B_12_ can be confounded by the administration of plasma and “normal” levels should be interpreted cautiously if drawn after initiating TPE. In case 4, a B_12_ level drawn after TPE was initiated was normal. However, the methylmalonic acid level was extremely elevated at 25,417 umol/l and ultimately diagnostic [14]. Patient age can help guide thinking because the median age of patients with B_12_ deficiency is 70 to 80 while two large series have shown that only fifteen percent of patients presenting with TTP are over the age of 60 years [15, 16].

A number of the pseudo-TTP case descriptions have suggested that very high levels of LDH are suggestive of B_12_ deficiency rather than TTP. Reports dating back 50 years have demonstrated unusually high levels of LDH in megaloblastic anemia, far in excess of that seen congenital and acquired hemolytic anemia. In two series of 27 and 16 patients with pernicious anemia, the mean LDH was 5360 IU/L and 3802 IU/L respectively with maximal values of 15,900 IU/L and 11,000 IU/L seen [17, 18]. These levels were much higher than those of the control groups with other types of hemolytic anemias. A reduction in LDH was observed after 4 to 6 days after B_12_ therapy. Accordingly, in 1961 Gronvall concluded *“Values of LDH exceeding 3000 U./ml./min. in anemia argue strongly for pernicious anemia”* [17]. The etiology of the high values was ultimately linked to ineffective erythropoiesis through LDH isoenzyme analysis [19].

The range of LDH levels seen in TTP case series is wide and partially overlaps with those reported for pseudo-TTP associated with B_12_ deficiency. The largest series are summarized as follows: In 36 patients with TTP seen at Washington University, the mean LDH was 939 IU/L, with a range from 328 to 28,000 IU/L. Thirty two of these 36 patients had an LDH of less than 2000 IU/L and 35 of 36 had LDH less than 3000 IU/L [13]. The single patient with LDH of 28,000 was found to have an ADAMTS13 level of 74 %. In 100 patients seen at the Cleveland Clinic with TTP, the mean +/− SD of LDH was 1338 +/− 945 IU/L [16]. Findings in 72 cases from Johns Hopkins showed a median LDH concentration of 1184 IU/L with an interquartile range of 152 to 5950 IU/L [20]. The 261 patients reported in the Oklahoma registry [21] were divided according to ADAMTS13 levels. For 201 patients with ADAMSTS13 levels equal to or above 10 %, the median LDH was 1090 (range 114 to 12,587), and for 60 patients with ADAMSTS13 levels below 10 %, the median LDH was 1407 (range 256 to 3909). Thus, it can be concluded that most patients with TTP will have LDH levels below 2500 IU/dl, but a minority of patients, including those with ADAMTS13 levels below 10 % (indicating bona fide TTP), have LDH values that exceed this threshold. Thus, an LDH value above 2500 IU/dl is seen infrequently in this disorder and should raise the possibility of pseudo-TTP associated with B_12_ deficiency.

The parameter that is most helpful in differentiating pseudo-TTP from TTP is the absolute reticulocyte count. The French series above noted reticulocytopenia in all seven patients reported. The reticulocyte counts in the individual cases were all inappropriately low for the level of anemia seen. The patient seen at our institution had a low corrected reticulocyte count of only 1.2 %. Thus, the absence of a brisk reticulocytosis should alert the provider to the possibility of nutritional deficiency driving the microangiopathy.

## Conclusions

Pseudo-TTP from B_12_ deficiency should be among the differential diagnoses of patients presenting with microangiopathic hemolytic anemia. Its resemblance to TTP is close enough that many of the reported cases have received TPE. It is possible that some of the cases in TTP series were actually pseudo-TTP given that it can present with a normal B_12_ level and normal MCV. Our literature review suggests that routine evaluation of suspected TTP cases should include a reticulocyte count, B_12_ and MMA level, and careful consideration of the differential diagnosis when the LDH level is above 2500 IU/L. Patient age should be considered given the wide difference between median ages at presentation of TTP and pseudo-TTP. Finally history of gastric bypass, while as yet unreported as a cause of pseudo-TTP, should alert the provider that B_12_ deficiency is a possible cause.

## Consent

Written informed consent was obtained from the patient for publication of this case report and any accompanying images. The consent was obtained in 2014, greater than one year following treatment and after complete recovery of her mental state. A copy of the written consent is available for review by the Editor of this journal.

## Abbreviations

ADAMTS13: a disintegrin and metalloproteinase with a thrombospondin type 1 motif, member 13
TPE: therapeutic plasma exchange
TTP: thrombotic thrombocytopenic purpura
